# Functional expression of dental plaque microbiota

**DOI:** 10.3389/fcimb.2014.00108

**Published:** 2014-08-14

**Authors:** Scott N. Peterson, Tobias Meissner, Andrew I. Su, Erik Snesrud, Ana C. Ong, Nicholas J. Schork, Walter A. Bretz

**Affiliations:** ^1^Infectious Diseases, J. Craig Venter InstituteRockville, MD, USA; ^2^Department of Molecular and Experimental Medicine at the Scripps Research InstituteLa Jolla, CA, USA; ^3^The Scripps Translational Science Institute and Scripps HealthLa Jolla, CA, USA; ^4^Department of Cariology and Comprehensive Care, College of Dentistry, New York UniversityNew York, NY, USA

**Keywords:** caries, oral microbiota, dental plaque, biofilm, transcriptome

## Abstract

Dental caries remains a significant public health problem and is considered pandemic worldwide. The prediction of dental caries based on profiling of microbial species involved in disease and equally important, the identification of species conferring dental health has proven more difficult than anticipated due to high interpersonal and geographical variability of dental plaque microbiota. We have used RNA-Seq to perform global gene expression analysis of dental plaque microbiota derived from 19 twin pairs that were either concordant (caries-active or caries-free) or discordant for dental caries. The transcription profiling allowed us to define a functional core microbiota consisting of nearly 60 species. Similarities in gene expression patterns allowed a preliminary assessment of the relative contribution of human genetics, environmental factors and caries phenotype on the microbiota's transcriptome. Correlation analysis of transcription allowed the identification of numerous functional networks, suggesting that inter-personal environmental variables may co-select for groups of genera and species. Analysis of functional role categories allowed the identification of dominant functions expressed by dental plaque biofilm communities, that highlight the biochemical priorities of dental plaque microbes to metabolize diverse sugars and cope with the acid and oxidative stress resulting from sugar fermentation. The wealth of data generated by deep sequencing of expressed transcripts enables a greatly expanded perspective concerning the functional expression of dental plaque microbiota.

## Introduction

Members of the oral microbial community play key roles in maintaining oral health and as putative agents responsible for the onset and progression of oral diseases. Previous studies have estimated that greater than 700 species of microorganisms inhabit the oral cavity (Moore and Moore, 1994; Darveau et al., 1997; Kolenbrander, 2000; Hutter et al., 2003). The application of high throughput, culture-independent metagenomics methodologies represents an approach that is well aligned with the high species diversity of oral microbiota. Our previous efforts to define the population structure of dental plaque microbiota revealed an impressive radiation of species derived from a substantially smaller set of genera (Peterson et al., 2011; Walter and Ley, 2011). The *Streptococcus* are dominant in dental plaque microbiota but include a variety of additional genera such as: *Veillonella, Campylobacter, Neisseria, Gemella, Granulicatella Capnocytophaga*, and *Fusobacterium*. A comparison of the saliva community composition of human subjects from China (Luo et al., 2012; Ling et al., 2013), the USA (Cephas et al., 2011) and the African continent (Nasidze et al., 2011) display a high level of variability. In each case the dominant genera identified are unique. The saliva microbiota of Chinese children featured a high proportion of Streptococcus (~40%) and Prevotella (~25%) and was complemented by 17 lower abundance genera (Ling et al., 2013). Despite the commonality of a plant-based diet, the saliva microbiota of human subjects from Sierra Leone (SL), the Democratic Republic of Congo (DRC), and the Batwa pygmies (BP) of Uganda display clear distinctions in community structure. *Streptococcus* spp. represented ~20% of the total in all groups. The SL saliva microbiota is dominated by *Enterobacter* spp. (~60%), whereas the subjects from the DRC displayed a high proportion of *Serratia* spp (~25%) and a relatively high abundance of *Rothia* spp. The other observed genera include taxa that are not significantly represented in saliva microbiota previously reported. These studies and others indicate that the microbiota may adopt a relatively large number of configurations in both health and disease (Cephas et al., 2011; Nasidze et al., 2011; Luo et al., 2012; Ling et al., 2013). The phylogenetic representation of related species in bacterial communities confer functional redundancy since their genomes encode a relatively high frequency of homologous protein functions. Such redundancy ensures that the loss of individual species within the community is functionally well tolerated and represents a likely basis of the high interpersonal variation observed in oral microbiota.

A variety of factors such as: genetic, immunological, behavioral, environmental, and mechanisms of vertical inheritance all play a role in defining the oral microbial community composition. Among these factors those pertaining to environment and particularly diet may be the most influential. In this manner, any case-control study attempting to relate microbial composition to features of the oral cavity in a state of health or disease is severely hampered by the fact that unrelated individuals participating in these studies do not share the same environment. The advantages of utilizing a twin study model, are numerous and importantly allow control over host genetics and relevant environmental factors, e.g., diet, vertical inheritance and lifestyle that serve to increase study power.

The healthy adult oral microbiota represents a highly tuned ensemble of species, selected for survival in a highly competitive and challenging environment that features frequent flux in dietary nutrients (Van der Hoeven and Camp, 1991), O_2_ concentration (Diaz et al., 2002), temperature (Fedi and Killoy, 1992), pH (Svensater et al., 1997), and energy metabolism (Palmer et al., 2006; Jakubovics et al., 2008). The dental plaque biofilm contains phylogenetically diverse acidogenic (acid-producers) species many that are also aciduric (acid-tolerant). Dental plaque biofilm-mediated sugar metabolism leads to the production of organic acids that reduce the pH of the biofilm microenvironment and represent key factor in the demineralization of the tooth surface (van Houte, 1994). The availability of dietary carbohydrates is key to biofilm initiation and development (Paes Leme et al., 2006). The production of acid may differentially inhibit resident microbial populations. Microbial metabolism of nitrogenous substrates has been attributed to the production of small arginine peptides that may elevate pH (Burne and Marquis, 2000). Likewise urease activity may also serve to elevate pH of the dental biofilm (Kleinberg, 2002). The dominance of the *Streptococci* and other members of the Firmicutes, dictate the overall fermentative activities in dental plaque.

The individual members of the dental plaque community are likely to belong to numerous and diverse functional networks. These networks may largely reflect cooperative activities of species to maintain environmental homeostasis. For example, the *Veillonella* exploit the metabolic activities of the dominant fermentative microbes. The *Veillonella* are asaccharolytic and derive energy from the metabolism of SCFAs (van der Hoeven et al., 1975; Noorda et al., 1988) producing shorter chain length acids with higher dissociation constants, thereby increasing the pH of the biofilm microenvironment. These acid sinks are critical to the growth and activity of the fermentative species. Interestingly, some acidogenic *Streptococcus* and *Granulicatella* encode the L-lactate dehydrogenase gene suggesting that they too may contribute to acid remediation of dental biofilms (McLean et al., 2012; Edlund et al., 2013).

In order to overcome the challenges associated with determining the species and functional activities of oral microbiota that maintain oral health or drive disease we must improve our understanding of how complex communities function and interact with one another. We have sampled the dental plaque of a large cohort of twin pairs in a longitudinal analysis spanning 3 years. Here we report on the transcriptional activity of the dental plaque microbiota of a twin cohort to improve our understanding of fundamental biochemical features of biofilm communities and the inter-relationships that exist between species in a feast or famine microenvironment.

## Materials and methods

### Dental caries phenotype determination

Dental caries examinations were performed on 38 subjects [19 twin pairs, 6 monozygotic (mz), 13 dizygotic (dz)]. The twin pairs were either concordant for dental health, C-F (*n* = 4 pairs), concordant for dental caries C-A (*n* = 6 pairs), or discordant for dental caries (*n* = 9 pairs). These subjects (5–7 years old) were medically healthy and presented with only primary dentition. This group of children resides in the suburbs of the city of Montes Claros, State of Minas Gerais, Brazil. Water fluoride levels in this city are less than optimal (<0.7 ppm) and dental check-ups for this group were negligible.

### Ethics statement

Parents signed informed consent approved by New York University and UNIMONTES (State University of Montes Claros) institutional review boards after the children assented.

### Dental caries examinations

We used a combination of three dental caries exams for accurate characterization of dental caries phenotypes in C-F and C-A subjects. These included: (1) *Clinical examination* of dental caries in all teeth, assessed with the aid of artificial light and a dental mirror according to NIDCR criteria (Kaste et al., 1996) to include white spot lesions and cavitated lesions; (2) *Digital imaging fiber-optic trans-illumination* (DIFOTI) recorded images of dental lesions (incipient and frank lesions) to complement the caries clinical examination (Schneiderman et al., 1997); (3) *Quantitative light fluorescence* (QLF) profiled images of dental lesions similar to the DIFOTI procedure that are not readily captured by visual examinations and complemented the caries clinical examination. C-A subjects had a range of 1–17 decayed tooth surfaces whereas C-F subjects presented with a decay component = 0. Caries-inactive (C-I) subjects presented with surfaces that had restorations provided in previous visits.

### Dental plaque biofilm sampling

Subjects were instructed to refrain from brushing or eating prior to sampling. Therefore, the subjects had not consumed a meal in at least 12 h prior to sample collection. Dental plaque samples were obtained using a sterile toothbrush passed slowly across all tooth surfaces. We elected to collect an overall plaque sample of the entire dentition rather than sampling site-specific surfaces that are associated with health or disease to enable characterizations that would otherwise be biomass limited. Moreover, our previous studies demonstrate that the dental microbiota associated with localized healthy tooth surfaces and caries lesions are similar within the same oral cavity (Corby et al., 2005). Dental plaque was dislodged from the toothbrush by agitation for 1 min into tubes containing 8 mL of sterile reduced transport fluid (RTF) (Syed and Loesche, 1972) held at 4°C prior to storage at −80°C.

### Bacterial mRNA isolation from dental plaque

Dental plaque samples were thawed and resuspended in RNAprotect reagent (Qiagen Inc) and stored at −80°C. RNA isolation was performed following the procedure recommended by the manufacturer for the mirVana RNA isolation kit (Ambion). The purified RNA was evaluated subjectively using the Agilent Bioanalyzer and quantitated using a UV spectrophotometer. We used hybridization-based subtraction methods to remove human and bacterial rRNA sequences from samples as described in detail http://www.hmpdacc.org/RSEQ/.

### RNA-Seq data analysis

We assembled a reference genome database comprised of 206 oral species (134 unique species groups) for read mapping. The RNA-Seq data was processed through a pipeline that performs a series of quality control steps. First, raw reads were examined using the FastQC (http://www.bioinformatics.babraham.ac.uk/projects/fastqc/) tool. Quality scores were calculated based on Illumina 1.5 encoding. Trimming of low quality base calls were conducted using Trimmomatic (http://www.usadellab.org/cms/?page=trimmomatic) by removing terminally located low quality bases (Phred scores <30) and cutting of reads when average quality dropped below 30. Finally, reads of 60 bases or less were removed from further analysis. The remaining reads were then evaluated for the presence of bacterial or human rDNA sequences using SortMeRNA (http://bioinfo.lifl.fr/RNA/sortmerna/) by filtering based on the default databases that include 16S, 23S, 18S, 5.8S, 5S, and 28S rRNAs. Sequences with similarity to these sequences are removed from further analysis. The remaining reads were then aligned to reference genomes using STAR aligner (https://code.google.com/p/rna-star/). In cases where sequence reads map to more than one location in a reference genome, or to more than one reference genome, the best alignment was selected. Sequence reads mapping sporadically and at a very low frequency to reference genomes (104 genomes) were dropped from further analysis.

### Gene expression analysis

Raw read counts from sequence alignment were assessed using htseq-count tool within the HTSeq python suite (http://www-huber.embl.de/users/anders/HTSeq/). For further analysis, the raw read counts were read into R/Bioconductor version 3/2.13 and were scaled using DESeq scaling factors (Anders and Huber, 2010) following log_2_ transformation (a constant +1 was added prior log_2_ transformation). Reads greater than 40 bases were used for mapping and 2 mismatches were allowed for mapping reads to reference genomes.

### Functional analysis

rRNA filtered reads were uploaded to the MG-RAST analysis platform for functional analysis (Meyer et al., 2008). Functional data from MG-RAST analysis was retrieved using matR package (https://github.com/MG-RAST/matR) and further analyzed using R/Bioconductor.

## Results and discussion

The majority of metagenomic analyses conducted thus far on the dental plaque microbiota have surveyed and compared the phylogenetic composition of communities associated with dental health and disease in the supra- and sub-gingival domains of the oral cavity. While informative, these studies do not provide insights into the functional features of these communities. Dental biofilms are comprised of metabolically active, metabolically inactive and dead cells. In order to evaluate the metabolically active cells of supragingival dental plaque biofilms we have conducted a survey of the RNA expression to gain insights of those functions that are important for survival and fitness in the highly competitive dental plaque biofilm. The species and relative contribution of transcripts to the transcriptome is largely consistent with our previous phylogenetic profiling results (Peterson et al., 2013) with respect to the genera and species present and their overall proportions within the community.

### Human cohort and RNA-Seq analysis

We performed RNA-Seq analysis of RNA isolated from dental plaque biofilms derived from 19 twin pairs. These subjects were given dental examinations that allowed each twin pair to be classified as belonging to one of three phenotypic classes (C-F, C-A, or C-I). RNA from each sample was subjected to RNA-Seq using the Illumina GSA platform to generate 100 base reads. An average of ~32.4 million reads/sample (range = 23–40 million) were generated. We created an oral cavity reference genome database consisting of a total of 206 reference genome sequences, representing 134 unique oral species. These sequences and the associated SOPs developed for microbial mRNA enrichment is available through http://www.hmpdacc.org/RSEQ/. Despite attempts to remove human and bacterial rDNA sequences, high levels of these sequences remained and were removed *in silico*. Table 1 summarizes the human cohort and bacterial sequences used for mapping. An average of ~55% of reads generated were readily mapped to HMP reference genomes emphasizing the overall relevance of selected genomes and utility of this community resource.

**Table 1:** **Human subjects and RNA-Seq statistics**.

**Subject**	**Caries status**	**Twin type**	**Filtered reads**	**Reads mapped**	**%**
2011	CA (1DS)	DZ	10404431	4690029	45.00
2012	CA (1DS)	DZ	5048821	2354837	46.64
2051	CF	MZ	15163860	6133805	40.45
2052	CF	MZ	8491819	3268038	38.49
2061	CA (2 DS)	DZ	5800355	2923616	50.41
2062	CF	DZ	4998861	2226509	44.54
2125	CA (17 DS)	MZ	5073718	2840183	55.98
2126	CA (6 DS, 3 FS)	MZ	10255337	5437838	53.03
2169	CF	MZ	5972974	3424681	57.34
2170	CA (1 DS)	MZ	7949869	4376808	55.05
2191	CF	MZ	6787165	3702907	54.56
2192	CF	MZ	5722083	2966892	51.85
2225	CF	MZ	7297864	3168223	43.42
2226	CI (1 FS)	MZ	9301393	2330247	25.06
2233	CF	DZ	4126376	1797372	43.56
2234	CF	DZ	5318936	2206502	41.48
2241	CA (1 DS)	DZ	3397928	1915754	56.38
2242	CA (1 DS)	DZ	4145884	2601872	62.76
2269	CA (2 DS)	DZ	3734615	2510588	67.23
2270	CF	DZ	3417500	2042613	59.77
2283	CI (2 FS)	DZ	5031738	3139713	62.40
2284	CA (1 DS)	DZ	4358907	2199217	50.45
2309	CF	DZ	9571270	3922570	40.98
2310	CF	DZ	7731245	2351980	30.42
2354	CA (1 DS)	DZ	4077295	2275040	55.80
2355	CA (1 DS)	DZ	1073838	464318	43.24
2930	CF	DZ	3873870	2279747	58.85
2931	CA (3 DS)	DZ	4071273	2225255	54.65
2954	CA (15 DS, 2 FS)	DZ	7338017	3247379	44.26
2955	CA (9 DS, 2 FS)	DZ	10133068	4293062	42.37
2991	CA (1 DS)	DZ	5331302	3270152	61.34
2992	CF	DZ	3267126	1265071	38.72
3214	CF	MZ	4008044	1758551	43.87
3215	CA (1 DS)	MZ	6357380	3360013	52.85
3306	CA (1 DS)	DZ	3773687	2163623	57.34
3307	CF	DZ	3748232	2198571	58.65
4131	CA (1 DS)	DZ	3656778	2075715	56.77
4132	CF	DZ	6734529	4223948	62.73

*DS, decayed surface; FS, filled surface*.

### Genetics, environment, and caries phenotype as determinants of transcriptional relatedness

**Figure 1:**
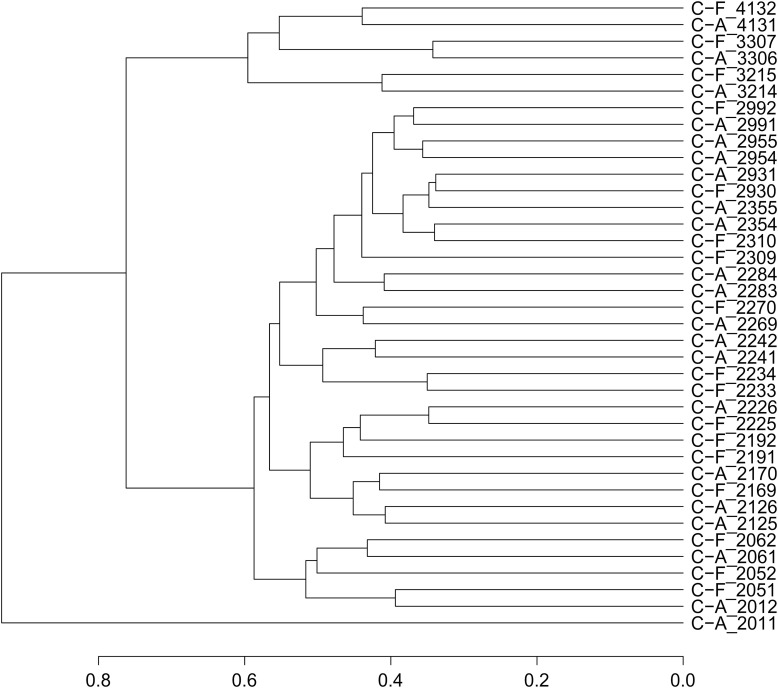
**Dendrogram of twin pairs based on RNA expression patterns**.

Based on the relative abundance and origin of the profiled transcripts, we generated a dendrogram of the samples to assess whether twin pairs clustered more tightly than unrelated individuals and whether caries phenotype altered those relationships (Figure 1). Fourteen of the 19 twin pairs were most similar to each other with respect to their gene expression patterns, suggesting that either genetic and/or environmental factors are significant determinants of dental plaque microbiota gene expression patterns. To evaluate the influence of host genetics on transcriptional profiles, we compared the linkage of MZ and DZ twin pairs. Among the six MZ twin pairs, four (66%), displayed linkage, compared to 10 of the 13 DZ twin pairs (76%). A comparison of the linkage relationships among discordant MZ and DZ twin pairs revealed that 100% of all discordant MZ (*n* = 3) and DZ (*n* = 6) twin pairs displayed linkage. These results suggest that genetic and/or environmental factors are dominant to caries status as determinants of gene expression patterns. This conclusion is supported by the finding that only 50% of the concordant twin pairs displayed linkage. The sample size evaluated here does not allow definitive conclusions with respect to the relative influence of genetic determinants compared to environmental factors. To achieve statistical support for these conclusions will require analysis of a larger number of twin pairs in longitudinal studies. The apparent lack of association between caries status and gene expression linkage in twin pairs must be interpreted with caution since linkage is dictated by global features of the transcriptome. It is expected that gene expression patterns that distinguish C-F and C-A subjects may involve the altered expression of a small fraction of the transcriptome and therefore would not be revealed by this analysis. In addition, some subjects went from a state of health to disease within a follow-up visit. It is possible therefore, that these subjects possessed a C-A signature at baseline, despite being clinically C-F. Detailed analysis of expression patterns to identify those genes that may clearly distinguish C-F and C-A subjects are ongoing and not reported here.

### Gene expression of genera present in dental plaque microbiota

**Figure 2:**
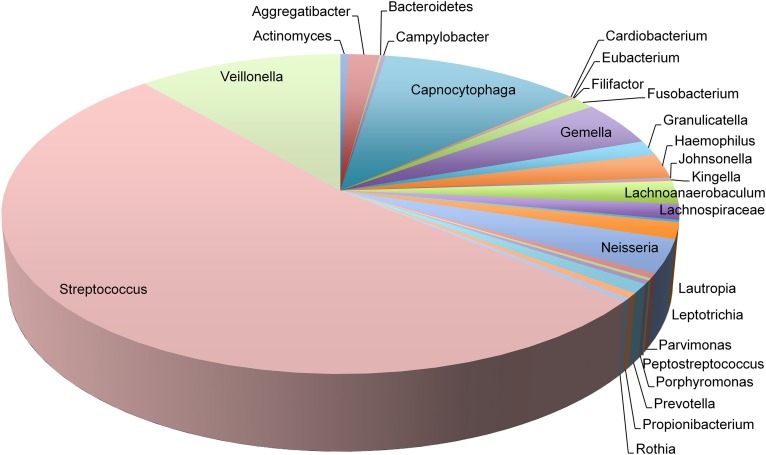
**Transcript abundance of representative genera in dental plaque biofilms**. Pie chart representing the proportions of transcripts mapping to 27 genera observed in 19 twin pairs.

In contrast to phylogenetic profiling of microbiota, RNA-Seq data permits the analysis of metabolically active members of the supragingival dental plaque biofilm. We detected transcripts mapping to 27 genera that spanned six orders of magnitude in abundance (Figure 2). Consistent with their numerical dominance in dental plaque, transcripts expressed by *Streptococcus* spp. were the most abundant (53% of total), nearly five times more than those expressed by the next most prevalent genera *Veillonella* spp. (11%) and *Capnocytophaga* spp. (11%). Transcripts from these genera together with *Gemella* spp. (5%) and *Neisseria* spp. (3%) comprised 83% of all mapped transcripts. Within individuals, additional genera contributed significantly to the dental plaque transcriptome (>1%) including: *Aggregatibacter* spp. (6 subjects), *Fusobacterium* (3 subjects), *Haemophilus* spp. (8 subjects), *Lachnoanaerobaculum* spp. (8 subjects), *Lachnospiraceae* spp (4 subjects), *Leptotrichia* (6 subjects), and *Parvimonas* spp. (1 subject). These findings underscore the interpersonal variation in the genera contributing to the dental plaque biofilm transcriptome. It will be of interest to correlate the 16S rDNA profiles of these subjects to determine the extent that transcript abundance is related to relative species abundance.

### Gene expression of species present in dental plaque microbiota

Several studies to date have surveyed the dental plaque biofilm and saliva microbiota using culture independent 16S rDNA sequencing (Lazarevic et al., 2010; Jiang et al., 2013; Yang et al., 2014). Attempts to define a core microbiome are challenging due to high interpersonal and geographic variability in microbiota composition and a strong shift toward the use of short read sequencing technologies that generally allow only genus level enumeration (Griffen et al., 2011; Li et al., 2013). The RNA-Seq data mapped to the reference genomes of 79 unique species. A significant number of reads mapped to 58 unique species in all subjects suggesting that they may represent a substantial fraction of a core dental plaque biofilm microbiota. It will be of interest to determine whether this core microbiome definition extends to additional geographies beyond the cohort examined here. Displayed as an aggregate, the transcripts expressed by individual species are relatively continuous over a broad abundance range (Figure S1). Transcripts derived from just 9 species including: *S. sanguinis* (16%), *S. mitis* (10%), *V. parvula* (9%), *Capnocytaphaga* sp. (9%), *S. oralis* (8%), *Streptococcus* sp. (7%), *G. haemolysans* (5%), *S. gordonii* (4%), and *Neisseria* sp. (3%) represented 71% of the dental plaque microbiota's transcriptome. An additional six species produced transcripts at 2% abundance. *S. mutans* was among these moderate abundance species. An additional 16 species produced transcripts at 1% of the total transcriptome. Together these 31 species account for 99% of the mapped transcripts observed in this cohort.

**Figure 3:**
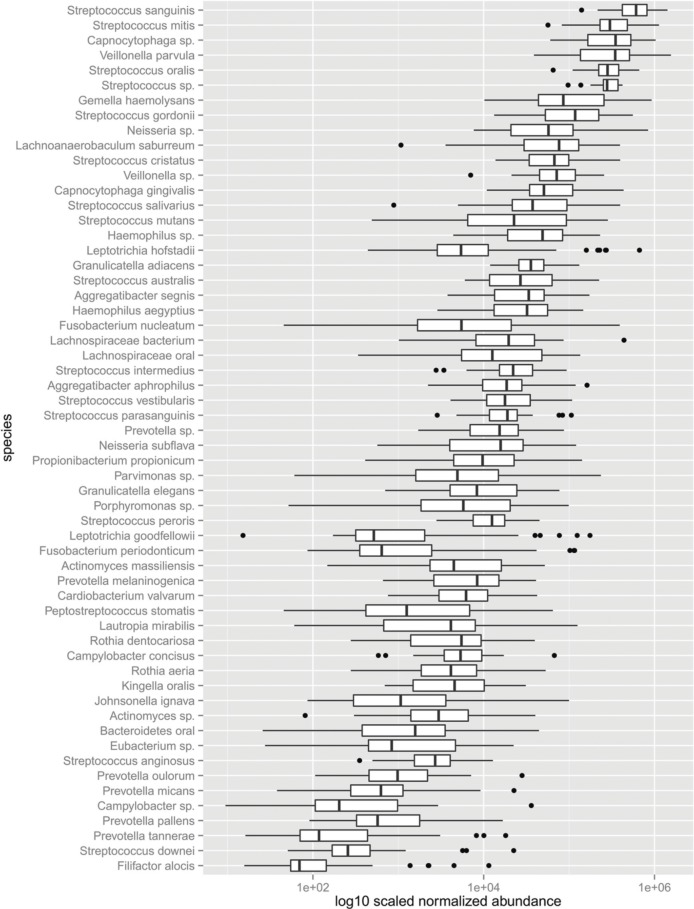
**Abundance of the dental plaque core microbiota**. Box and whisker plot. Outliers shown as dots represent values 1.5 times great or less than the upper and lower quartile, respectively.

The frequency of observed transcripts expressed by individual species also spanned six logs in magnitude (Figure 3). The abundance of transcripts generated by individual species is variable across subjects, varying by approximately two logs or less. The variation in transcript abundance across human subjects is consistent with numerous reports describing the high interpersonal variation in phylogenetic representation of supragingival dental plaque (Bik et al., 2010; Gross et al., 2010). *Streptococcus* spp., with the exception of *S. mutans* displayed rather limited variation in transcript abundance across subjects, whereas other species display markedly increased variability. The transcripts expressed by *Fusobacterium nucleatum* were detected over a range of 4 logs. Approximately 40% of the species displayed extreme transcript abundance variation (>2 SD from the mean). It is notable that many of the outliers tend to be over-represented with respect to the mean as illustrated by those expressed by *Leptotrichia hofstadii*, suggesting that in some instances the abundance of these species may be highly over-represented in subjects. The transcript abundance generated by species, of moderate and low abundance display the largest variation. These results provide a set of parameters that define fluctuations in phyla as it relates to health and disease states. It is evident from these results that species-specific transcriptional variability may range from biologically significant to inconsequential.

### Functional networks based on gene expression correlations

**Figure 4:**
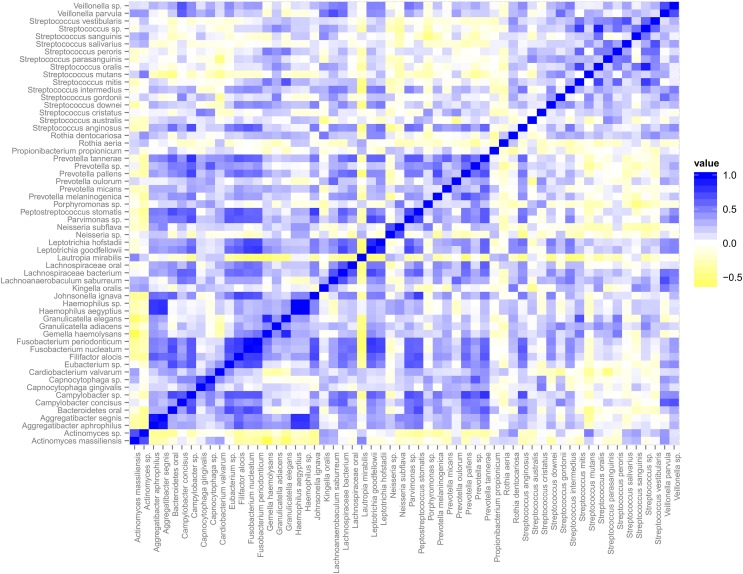
**Spearman Correlation matrix of the dental plaque core microbiota**. Blue indicates positive correlation and yellow indicates negative correlation.

The relative abundance of species within the dental plaque biofilm community is dictated by numerous and mostly undefined signals present in the microenvironment. These signals are both host and microbe-generated. The cooperative and antagonistic relationships amongst resident species in dental plaque biofilms suggest that the abundance of individual species and their transcripts is not independent of the activities of other species (Tong et al., 2008; Tamura et al., 2009). This speculation is strongly supported by the data. We used Spearman correlation to address whether the transcriptional activities of individual taxa display relationships (Figure 4). Correlations based on transcript abundance across subjects indicate that the majority of correlations amongst genera are positive. Multiple *Streptococcus* spp. display weak positive correlations with one another. This relationship may reflect the large overlap in sugar utilization potential encoded by these genomes that provide broad similarities in environmental conditions that co-select for increased growth and/or metabolic activity. This trend is evident for a number of other genera wherein member species display correlated transcriptional activity. Exceptions included: *Gemella* spp. (*G. elegans* and *G. adjacens*) and *Rothia* spp. (*R. aeria* and *R. dentocariosa*) that do not appear significantly correlated. *Actinomyces* spp. and *Lautropia mirabilis* display transcriptional activity that is largely anti-correlated with the majority of the dental plaque community, suggesting that the signals favoring their metabolic activity may be distinct compared to the majority of the dental plaque microbiota.

**Figure 5:**
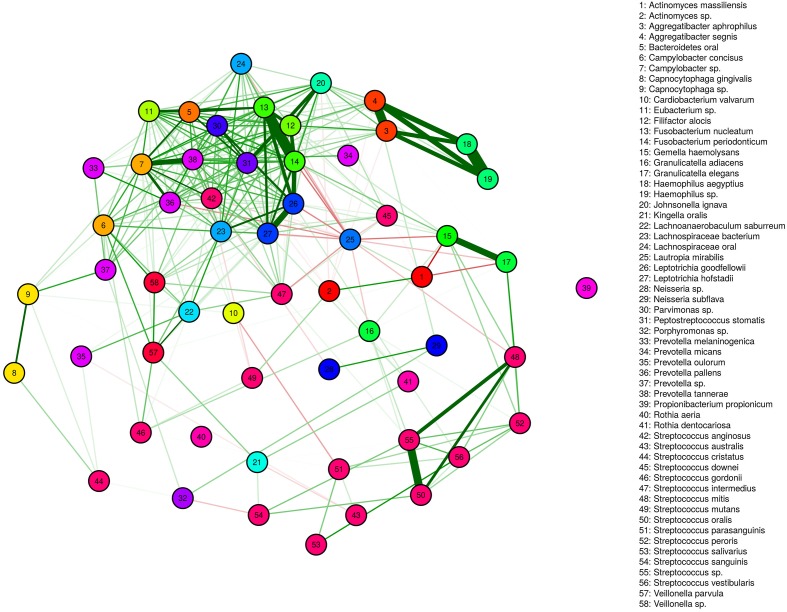
**Functional Networks**. Positively correlated (green) and anti-correlated (red) transcripts define networks. The thickness of lines indicates the strength of the correlation.

We identified several genera/species displaying both correlated and anti-correlated transcriptional activity. These relationships define functional networks representing a spectrum of simple and complex community relationships (Figure 5). A deeply integrated network involving, predominantly positive interactions are evident. It is interesting that this high-density network is highly diverse in its membership and includes more than half of the observed genera. The high-density cluster of interactions is relatively devoid of *Streptococcus* spp. Somewhat surprising, the *Streptococcus* display a range of mostly weak positively correlated networks that are relatively independent of the expression patterns of the majority of the microbial community. High interpersonal and geographic variation of dental plaque microbiota has hampered our ability to identify the microbial signatures associated with dental health and disease. The network relationships observed suggest that the fluctuations of single species are in many instances likely to be accompanied by shifts in other species in the network. These functional networks represent a potentially simplifying framework and may represent a more effective way to compare features of microbiota associated with health and disease. It is possible that genera unique to particular geographies may belong to the functional networks observed and described below.

**Figure 6:**
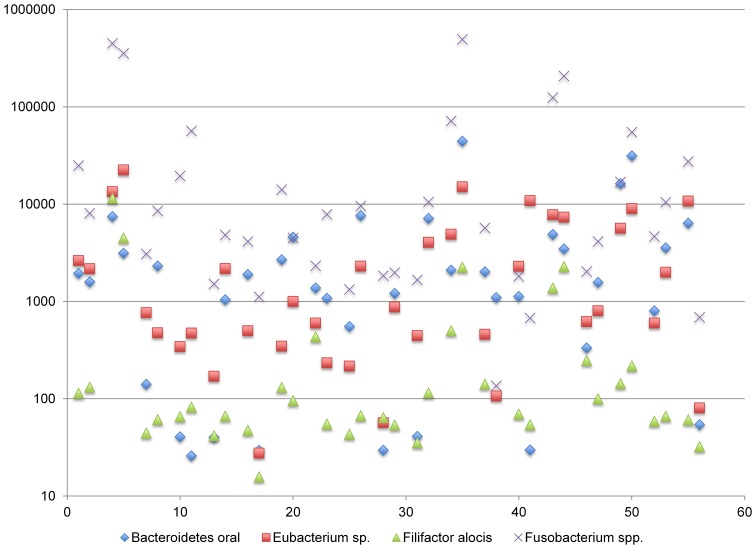
**Functional Network in dental plaque microbiota, complex I**. The y-axis displays read counts on a log scale. The x-axis displays subjects in the order shown in Table 1.

One positively correlated network includes the genera *Bacteroides*, *Eubacterium*, *Filifactor*, and *Fusobacterium* (complex I). The abundance of transcripts generated by these genera span approximately two logs and vary across subjects in a coordinated manner (Figure 6). With some exceptions, the *Fusobacterium* are the most abundant genera within this network, whereas the remaining genera are more variable in relative abundance. The species membership of this network includes: *F. nucleatum, Fusobacterium periodonticum, B. oral* an uncharacterized *Eubacterium* spp., and *Filifactor alocis*. The mixed dominance relationships of this network across subjects may suggest that the signals that regulate the growth behavior are complex.

Another positively associated network includes the genera *Peptostreptococcus* spp., *Bacteroides* spp., *Campylobacter* spp., *Johnsonella* spp., and *Parvimonas* spp. (complex II) The relative abundance of this network is more tightly linked, compared to complex I. The variation in abundance across subjects is slightly more than 1 log (Figure S2). However, the dominance relationships of complex II are more variable. The species comprising this network are *Peptostreptococcus stomatis, Bacteroidetes oral, Campylobacter concisus*, an uncharacterized *Campylobacter* spp., *Johnsonella ignava*, an uncharacterized *Parvimonas* spp., *Lachnospiraceae bacterium*, and *Lachnospiraceae oral*. The observed fluctuations are relevant in that certain configurations drive the abundance of *Parvimonas* and other genera into an abundance range that may be of biochemical consequence to the microenvironment.

**Figure 7:**
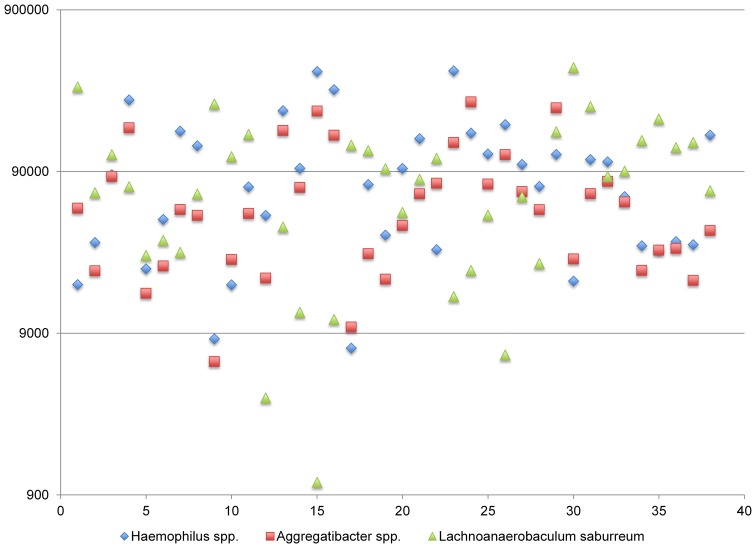
**Functional Network in dental plaque microbiota, complex III**. The y-axis displays read counts on a log scale. The x-axis displays subjects in the order shown in Table 1.

A third network including: *Haemophilus* spp., *Lachnoanaerobaculum* spp., and *Aggregatibacteria* spp. (complex III) display both positive and negative correlations (Figure 7). The abundance of transcripts expressed by *Haemophilus* spp. and *Aggregatibacteria* spp. are tightly linked, generally differing by less than 5-fold. By contrast, the abundance of *Lachnoanaerobaculum* spp. is anti-correlated with respect to these genera. When *Haemophilus* spp. and *Aggregatibacteria* spp. transcript levels are high, *Lachnoanaerobaculum* spp. transcript levels are low and *vice-versa*. Inspection of Figure 5 shows that *Aggregatibacteria* spp. are positively correlated with the high density functional network, whereas the *Haemophilus* spp. is only indirectly linked to the large network, based on its strong positive interactions with *Aggregatibacteria* spp. The behavior of this complex may be the result of specific niche associated signals that favor the outgrowth of one group and reciprocally inhibit the other. It is of potential interest that members of this complex “aggregate” at a point where transcripts generated by each member genera are in the range of 0.5% of the total. It is unclear whether this aggregation point has biological significance or represents a point of complex equilibrium, the balance of which can be disturbed in predictable ways. The species involved in this network are *H. aegyptus* and an uncharacterized *Haemophilus* spp. and two Aggregatibacter spp., *A. segnis* and *A. aphrophilus*.

The genera: *Leptotrichia* spp., *Lautropia* spp., and *Lachnospiraceae* spp. define another network involving positive and negative correlations (Figure S3). The transcript abundance generated by *Leptotrichia* spp. and *Lachnospiraceae* spp. is positively correlated and anti-correlated with respect to *Lautropia* spp. (complex IV). An analysis of these correlations at the species level revealed the network members to include: *L. hofstadii, L. bacterium, L. oral, Leptotrichia goodfellowii*, and *L. mirabilis*. Referring to Figure 5, we see that *L. mirabilis* is conspicuous in its nearly exclusively anti-correlated relationships. The majority of the anti-correlated relationships involve many of the genera making up the high-density network. The *Lachnospiraceae* are generally dominant in this network, although relatively frequent co-dominance with *Leptotrichia* are observed. One subject, showed *Lautropia* dominance and was associated with uncharacteristically low abundance of transcripts expressed by *Lachnospiraceae* and *Leptotrichia*.

A fifth functional network consisting of the genera *Streptococcus* spp., *Parvimonas* spp., *Eubacterium* spp. and *Campylobacter* spp. (complex V) was noted (Figure S4). The transcripts produced by these genera are positively correlated but anti-correlated with *Streptococcus* spp. The species involved in this network include: *Campylobacter concisus*, and an uncharacterized *Campylobacter* spp., *Parvimonas* spp. *Eubacterium* spp. It was difficult to identify any species within the *Streptococcus* that exhibited uniform anti-correlated transcription suggesting that the growth inhibiting influence of the *Streptococcus* within this network likely involves the combined activities of two or more species. It may be speculated that the overall balance between the *Streptococcus* and the remainder of the complex is based on sugar availability, since the fermentative *Streptococcus* may thrive under conditions that differ from the remainder of the complex that are primarily asaccharolytlic (*Campylobacter* spp., *Parvimonas* spp. *Eubacterium* spp.). Despite various reports of antagonistic relationships among *Streptococcus* spp., we do not observe such anti-correlated relationships at the level of transcription. The transcripts produced by the two most abundant species, *S. sanguinis* and *S. mitis* are positively correlated, however it is of potential interest that in instances where *S. mitis* is numerically dominant to *S. sanguinis*, the level of the *S. sanguinis* transcripts are reduced, suggesting that *S. mitis* may directly inhibit *S. sanguinis* when it is the most abundant species present.

An additional anti-correlated network (complex VI) including the numerically dominant genera *Gemella* spp. are strongly anti-correlated with those produced by *Actinomyces* spp. (Figure S5). From this data it is not clear, whether the conditions promoting high transcriptional activity of the *Gemella* spp. is inhibitory to *Actinomyces* spp., or if conversely the conditions selecting for elevated transcriptional activity of *Actinomyces* is inhibitory to *Gemella* spp. When analyzed at the species level, we identified *A. massiliensis* and *G. haemolysans* as the members of this network.

### Functional analysis of dental plaque biofilm gene expression

**Figure 8:**
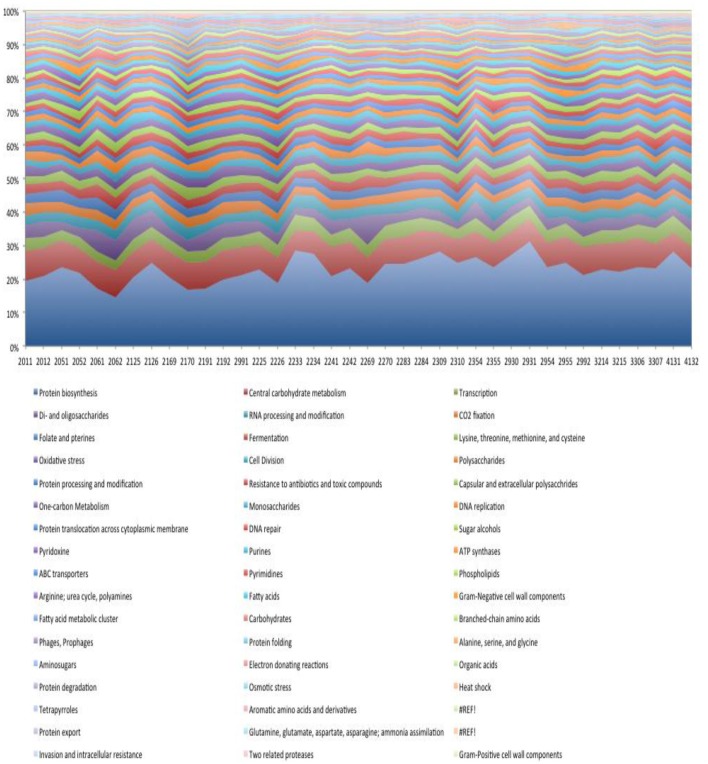
**Functional role categories expressed in dental plaque biofilm**.

Using the SEED subsystems role categories within the MG-RAST metagenomic analysis tool we see that despite the relatively large interpersonal variation in species-specific transcription, the representation of functional role categories is more homogeneous across subjects (Figure 8). This result is consistent with previous reports based on the analysis of functional annotation of metagenomic DNA sequences (Human Microbiome Project, 2012). The breadth of functions expressed at >1% of the total is substantial and descriptions of each are beyond the scope of this manuscript. A detailed analysis of these functions is ongoing. The most abundant and variable functional role category involves transcripts encoding functions pertaining to protein translation (range = 13–28%). The most abundant transcripts expressed by the dental plaque microbiota encode ribosomal subunit biogenesis (8.9% of total), and transcripts derived from the translation elongation factors, EF-Tu and EF-G (4% of total). The next most transcriptionally abundant functional category relates to carbohydrate utilization (~10% of total). The functions contributing to this role category include those involved in glycolysis/gluconeogenesis and the Entner-Doudoroff pathway that convert glucose to pyruvate. Transcripts encoding RNA polymerase subunits were also prevalent.

Transcripts encoding functions related to monosaccharide and disaccharide metabolism represent a significant portion of the dental biofilm transcriptome (~15% of total). Transcripts encoding enzymes for the metabolism of allose, galacuronate/glucuronate, gluconate, ribose, sorbitol/sorbose, tagatose/galctitol, fucose, rhamnose, and xylose were observed. In most subjects the transcripts encoding enzymes for the metabolism of tagatose (a stereoisomer of fructose) and galactitol (generated by the metabolism of lactose and subsequent conversion to galactose) were the most highly represented, although in three subjects the dominant transcripts in this category encoded enzymes involved in sorbitol (the sugar alcohol form of glucose) and sorbose metabolism. In general, transcripts encoding di-saccharide metabolism were more prevalent than those encoding monosaccharides by a factor of ~2. The most abundant transcripts associated with di-saccharide metabolism, encoded functions involved in lactose and galactose metabolism, although in a few subjects, transcripts encoding trehalose metabolism functions were the most abundant in this category.

We observed a relatively large number of transcripts encoding functions related to the Calvin Benson cycle, a CO_2_ fixation pathway used in plants. The vast majority of reads (74%) related to this pathway corresponded to GAPDH, fructose-bisphosphate aldolase (9%), phosphoglycerate kinase (5%), triose phosphate isomerase (2%) likely reflecting the glycolytic pathway. Other transcripts encoding transketolase, ribose-5-phosphate isomerase, ribulose phosphate 3-epimerase, participate in the pentose phosphate pathway. Therefore, the reads assigned to the Calvin Benson cycle in dental plaque microbiota samples represent enzymes with overlapping functions in glycolysis and the pentose phosphate pathway. The transcripts encoding for sugar alcohol utilization were predominantly associated with ethanolamine utilization (~50% of total) and mannitol utilization (~25% of total).

### Oxidative stress

The dental plaque microbiota produces a substantial number of transcripts encoding stress response adaptations including detoxification, heat shock, osmotic and oxidative stress. The majority of stress response transcripts (50–75% of total) were associated with oxidative stress suggesting that oxidative stress may be the dominant stressor of dental biofilm microbial communities (~2% of all transcripts). Two inter-related systems are dominant within this group, transcripts encoding superoxide dismutase, that mediate the conversion of superoxide to molecular O_2_ and H_2_O_2_. These transcripts represented 22% of those related to oxidative stress and 0.4% of the total transcriptome. It is interesting to note that this reaction is H^+^ consuming and given its relative abundance may play a role in acid remediation in dental plaque. An impressive number of transcripts are produced by dental plaque biofilm encoding functions that serve to metabolize (detoxify) superoxides and peroxides. Peroxiredoxins (9% of oxidative stress transcripts, 0.2% of total transcriptome) alter their redox state in order to convert H_2_O_2_ to H_2_O. This enzyme activity represents a potential biomarker of squamous cell carcinomas (Huang et al., 2011; Jancsik et al., 2013). Transcripts encoding catalase (1% of oxidative stress genes) performs the same conversions in the biofilm community using an Fe redox process. Interestingly, another prominent set of transcripts encoding ferroxidase (4% of oxidative stress genes), an enzyme that reduces Fe^2+^ to Fe^3+^ while consuming H^+^. Ferritin-like proteins also have ferroxidase activity and were expressed at similar levels as ferroxidase. The relevance of this pathway is based on the damaging effects of ferrous Fe^2+^ in the presence of H_2_O_2_ on Fe-S cluster containing proteins.

### Resistance to antibiotics and toxic compounds

The transcripts encoding resistance to antibiotics and toxic compounds was of interest. The majority of transcripts encode functions pertaining to metal tolerance and regulation. Large and diverse systems devoted to the maintenance of metal homeostasis underscores the relative importance of these systems. More than 16% of transcripts within this group encode mercury (II) reductase (EC 1.16.1.1). This enzyme mediates the generation of NADPH accompanied by the reduction of Hg to Hg^2+^. Copper homeostasis is maintained by P-type ATPases that use cellular energy (ATP) to pump Cu^2+^ ions out of the cell. These systems are greatly expanded compared to those functions related to other metals and toxins including cadmium, cobalt and arsenic. Transcripts involved in antibiotic resistance were difficult to interpret since many transcripts assigned to this group pertain to proteins that when mutated confer resistance to antimicrobial drugs. However, transcripts encoding the acriflavin resistance complex (AcrA and AcrB), that confers protection to cells from hydrophobic inhibitors including many common antibiotics in use today were prevalent. Transcripts corresponding to this multi-drug efflux system were 4% of the total in this group. Additionally, 1.6% of transcripts within this group encode a putative macrolide-specific efflux system. Transcripts encoding for proteins involved in acid stress and bacteriocins represented only minor components of the dental plaque biofilm's transcriptome across all subjects, including those with high caries activity.

## Summary

The results presented here have provided a number of unique insights with regard to the biochemical priorities and the environmental and/or genetic influence on these patterns of the dental plaque biofilm microbiota. Gene expression patterns amongst some genera are coordinated. Previous studies have attempted to recognize associations between features of the microbiota (individual genera or species) and dental health/disease. These studies have been confounded by an inability to control genetic and environmental factors, high interpersonal and geographical variation of dental biofilm communities. The recognition of functional networks operational in dental plaque communities may be of importance since it reduces the number of independent variables that may define dental health and caries activity. A longitudinal study of these networks as human subjects transition from C-F to a C-A phenotype will provide a direct test of the biological significance of these networks. The dental biofilm microbiota devotes a significant amount of its transcriptional potential to the expression of proteins that substantially remediate superoxides and peroxides and H^+^ produced by fermentative bacterial species. Maintenance of metal homeostasis, particularly of Fe^2+^ that are damaging to Fe-S cluster containing proteins in the presence of H_2_O_2_, also uses biochemical processes that consume H^+^. These important stress response pathways may represent a previously overlooked system used by dental biofilm microbiota to cope with a low pH microenvironment.

### Conflict of interest statement

The authors declare that the research was conducted in the absence of any commercial or financial relationships that could be construed as a potential conflict of interest.
