# Supplementary material for: Functional expression of dental plaque microbiota
Source: Front Cell Infect Microbiol. 2014 Aug 14;4:108. doi: 10.3389/fcimb.2014.00108 (PMC4132376; doi:10.3389/fcimb.2014.00108)
Supplement: Supplementary file 1 [file DataSheet1.DOCX]

|  |
| --- |
| **Supplementary Figure 1.** Summed transcript abundance expressed by individual species comprising a  core dental plaque biofilm. |

|  |
| --- |
| **Figure S2. Functional Network in dental plaque microbiota, complex II.** The  y-axis displays read counts on a log scale. The x-axis displays subjects in the order  shown in **Table 1.** |

|  |
| --- |
| **Figure S3. Functional Network in dental plaque microbiota, complex IV.** The y-axis  displays read counts on a log scale. The x-axis displays subjects in the order shown in **Table 1.** |

|  |
| --- |
| **Figure S4. Functional Network in dental plaque microbiota, complex V.** The y-axis  displays read counts on a log scale. The x-axis displays subjects in the order shown in **Table 1.** |

|  |
| --- |
| **Figure S5. Functional Network in dental plaque microbiota, complex VI.** The y-axis  displays read counts on a log scale. The x-axis displays subjects in the order shown in **Table 1.** |
